# An Immune Atlas of Nephrolithiasis: Single-Cell Mass Cytometry on SIRT3 Knockout and Calcium Oxalate-Induced Renal Injury

**DOI:** 10.1155/2021/1260140

**Published:** 2021-11-20

**Authors:** Wei Zhang, Ling Li, Ti Zhang, Xiaomin Gao, Zeyu Wang, Shaoxiong Ming, Ziyu Fang, Min Liu, Hao Dong, Baoyi Zhu, Junhao Liao, Jianwen Zeng, Yonghan Peng, Xiaofeng Gao

**Affiliations:** ^1^Department of Urology, Changhai Hospital, Naval Medical University, Shanghai 200433, China; ^2^National Clinical Research Center of Kidney Diseases, Jinling Hospital, Nanjing University School of Medicine, Nanjing, 210002 Jiangsu, China; ^3^Department of Urology, The Sixth Affiliated Hospital of Guangzhou Medical University, Qingyuan People's Hospital, Qingyuan, 511518 Guangdong, China

## Abstract

**Background:**

As a common urological disease with a high recurrence rate, nephrolithiasis caused by CaOx may elicit a strong immunologic response. We present a CyTOF-based atlas of the immune landscape in nephrolithiasis models to understand how the immune system contributes to, and is affected by, the underlying response caused by SIRT3 knockout and CaOx inducement.

**Materials and Methods:**

We performed a large-scale CyTOF analysis of immune cell abundance profiles in nephrolithiasis. The immunophenotyping data were collected from four different mouse models, including the SIRT3 wild-type or knockout, including and excluding CaOx inducement. Unsupervised analysis strategies, such as SPADE and viSNE, revealed the intrarenal resident immune components and the immune alterations caused by SIRT3 knockout and CaOx-induced renal injury.

**Results:**

An overview analysis of the immune landscape identified T cells and macrophages as the main immune cell population in nephrolithiasis models. Highly similar phenotypes were observed among CD4^+^ and CD8^+^ T cell subsets, including cells expressing Ki67, Ly6C, Siglec-F, and TCR*β*. Macrophages expressed a characteristic panel of markers with varied expression levels including MHC II, SIRP*α*, CD11c, Siglec-F, F4/80, CD64, and CD11b, indicating more subtle differences in marker expression than T cells. The SIRT3^KO^/CaOx and SIRT3^WT^/CaOx groups exhibited global differences in the intrarenal immune landscape, whereas only small differences existed between the SIRT3^KO^/CaOx and SIRT3^KO^/Ctrl groups. Among the major immune lineages, the response of CD4^+^ T cells, NK cells, monocytes, and M1 to CaOx inducement was regulated by SIRT3 expression in contrast to the expression changes of B cells, DCs, and granulocytes caused by CaOx inducement. The panel of immune markers influenced by CaOx inducement significantly varied with and without SIRT3 knockout.

**Conclusion:**

In a CaOx-induced nephrolithiasis model, SIRT3 has a critical role in regulating the immune system, especially in reducing inflammatory injury. The characteristic panel of altered immune clusters and markers provides novel insights leading to improved prediction and management of nephrolithiasis.

## 1. Introduction

Nephrolithiasis is a common urological disease with a nearly 50% recurrence rate within five years of the first occurrence. It has been reported that 80% of stones associated with the disease are composed of calcium, occurring as pure calcium oxalate (CaOx) or mixed with calcium phosphate (CaP) [1]. The formation of nephrolithiasis starts with supersaturation and crystallization of CaOx in the renal tubular lumen [2]. The deposit of CaOx crystals adheres to injured and dead renal tubular epithelial cells, leading to further crystal aggregation [3]. Therefore, necrosis and apoptosis of renal tubular epithelial cells are important in CaOx kidney stone formation.

Sirtuin 3 (SIRT3) is an NAD-dependent deacetylase that regulates acetylated substrate peptides, maintains energy homeostasis, and decreases ROS production and inflammation in proximal tubular epithelial cells [4]. Previous studies have shown that SIRT3 could prevent oxalate damage by promoting M2 polarization of macrophages and inhibiting cell death in renal tubular epithelial cells, indicating that SIRT3 has a protective role in the pathophysiology of nephrolithiasis [5].

Emerging data suggest that urinary CaOx could elicit a strong immunologic response. Kusmartsev et al. demonstrated that renal CaOx crystal deposits are usually surrounded, engulfed, and eventually disintegrated by tissue macrophages [6]. Furthermore, these activated macrophages release an array of cytokines and chemokines to attract circulating monocytes to the site of the injured tissue [7]. Similarly, Taguchi et al. recently demonstrated by renal papillary tip tissue biopsy that human CaOx stone formers have high amounts of tissue inflammatory markers [8]. However, macrophages may exhibit both proinflammatory and anti-inflammatory functions, depending on their phenotype and activation status.

Innovative single-cell technologies such as mass cytometry (CyTOF) can be used to comprehensively analyze the immune behavior in nephrolithiasis. In particular, CyTOF enables the simultaneous measurement of parameters previously presented at the single-cell level by combining metal isotope-labeled antibodies with mass spectrometry detection [9]. CyTOF has not been used in nephrolithiasis, which is associated with extensive immune alterations. In this study, we present a CyTOF-based atlas of the immune landscape in nephrolithiasis models. Unsupervised analysis strategies, such as SPADE and viSNE, are used to reveal the intrarenal resident immune components and the immune alterations caused by SIRT3 knockout and CaOx-induced renal injury.

## 2. Materials and Methods

### 2.1. Animal Models of Renal Injury

All animal experiments were performed in accordance with the Guidelines for the Care and Use of Laboratory Animals of the Laboratory Animal Ethics Committee of the Naval Medical University. SIRT3 knockout C57BL/6 mice were purchased from Shanghai Model Organisms Center, Inc. (Shanghai, China) and bred in an experimental animal room demonstrated to be specific-pathogen-free (SPF). For nephrolithiasis model establishment, mice received an intraperitoneal injection of 120 mg/kg glyoxylic acid (TCI Shanghai) for seven consecutive days, according to published protocols [10, 11]. Each mouse received 200 *μ*l of glyoxylic acid solution, which was made by dissolving 900 *μ*l of glyoxylic acid in 50 ml of 0.9% saline. Mouse kidneys were harvested 24 h after the last injection.

### 2.2. CyTOF Analysis of Immune Cells

All the kidney samples were processed in the same batch. CyTOF analysis was performed by PLTTech, Inc. (Hangzhou, China) according to a published protocol [12]. The kidney tissue was dissociated into a single-cell suspension with a mixture of DNase I, collagenase IV, and hyaluronidase (Sigma-Aldrich). Immune cells were enriched using Percoll density gradient media (Sigma-Aldrich), and erythrocytes were fully removed using ACK Lysing Buffer (Sigma-Aldrich). Qualified samples were blocked and stained with a mixed panel of surface antibodies, followed by overnight fixation. After fixing with Fix & Perm Buffer (Fluidigm), the cells were incubated in an intracellular antibody mix. The signals of the stained cells were detected using a CyTOF system (Fluidigm). The types of immune cells were identified via nonlinear dimensionality reduction (t-SNE), followed by density clustering.

### 2.3. Statistical Analysis

The Mann–Whitney *U* test was used to compare the differences between the two test groups. One-way ANOVA was used to determine the differences among the three or four groups. Statistical significance was set at *P* < 0.05.

## 3. Results

### 3.1. Immunophenotyping of Nephrolithiasis Models Using Mass Cytometry

**Figure 1:**
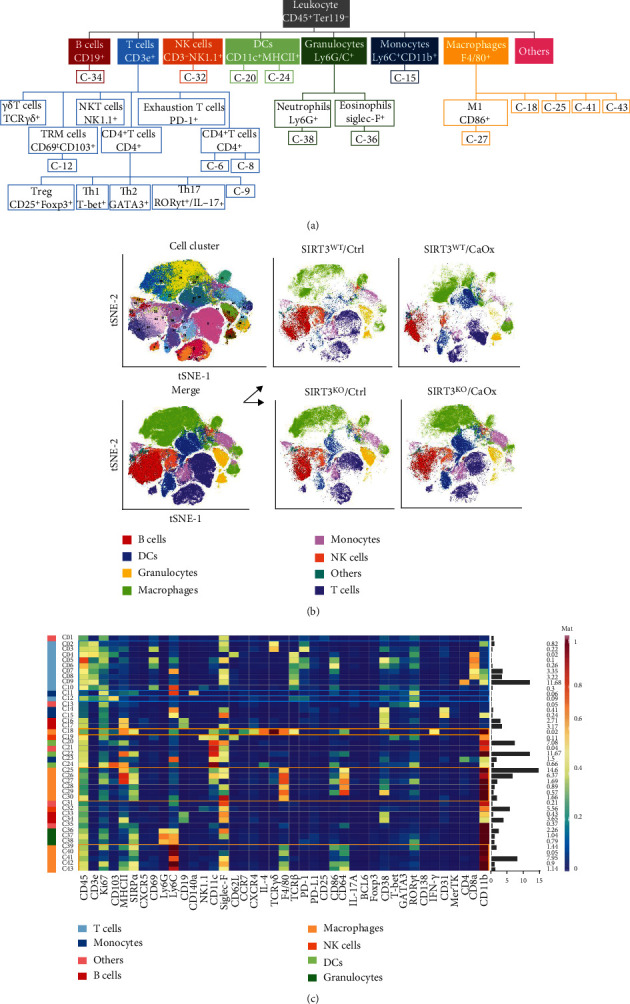
Characterization of immune cells in nephrolithiasis using mass cytometry. (a) Schematic showing how clusters are related to parent populations. (b) t-SNE maps displaying 9935 cells from the nephrolithiasis models analyzed with 42-antibody panel and colored by the main cell populations. (c) Heatmap showing normalized expression of the markers from 42-antibody panel for PhenoGraph clusters. Clusters are grouped by expression profiles, and cell types are indicated by color. The cluster IDs and relative frequencies are displayed as a bar graph on the left.

We performed a large-scale CyTOF analysis of immune cell abundance profiles in nephrolithiasis. Briefly, the immunophenotyping data were collected from four different mouse models, including SIRT3 wild-type or knockout, CaOx inducement, or noninducement (Supplementary Figure 1A). There were 5, 4, 5, and 5 mouse, respectively, in SIRT3^WT^/Ctrl, SIRT3^WT^/CaOx, SIRT3^KO^/Ctrl, and SIRT3^KO^/CaOx groups. To confirm the efficiency of SIRT3 knockout and CaOx inducement, we performed von Kossa staining for calcium deposits and immunohistochemical staining for SIRT3, cleaved caspase-3, and BCL-2 in the kidney (Supplementary Figure 1B). Von Kossa staining revealed that SIRT3 knockout increased calcium deposits in the kidney, especially in the renal cortex. Consistently, immunohistochemical staining indicated that CaOx-induced renal injury was significantly enhanced by SIRT3 knockout. These results confirmed that high expression of SIRT3 was required to maintain renal homoeostasis and protect against CaOx-induced cell death. We stained cells with a 42-antibody panel to identify different populations of naive, memory, effector, regulatory, and exhausted T cells, B cells, NK cells, monocytes, macrophages, DCs, and granulocytes (Figure 1(a)). A summary of the 42-antibody cocktail and output data from our mass cytometry experiments, including the total number and viability of cells collected for each sample, event counts detected by the instrument, and number of lives for analysis, is provided in Supplementary Figure 1C and 1D.

### 3.2. Overview Analysis of Immune Landscape in Nephrolithiasis Models

To partition the cells into distinct phenotypes, the PhenoGraph clustering algorithm was applied to illustrate the phenotypic adjacency of cells in a high-dimensional space. This analysis identified the main immune cell types (Figures 1(b) and 1(c)). Macrophages, T cells, and DCs accounted for the majority of the immune cell population in the nephrolithiasis models, respectively, with a mean of 27.7 ± 6.7%, 21.8 ± 4.4%, and 13.7 ± 8.2%, respectively. The mean frequencies of B cells, monocytes, and NK cells were 12.8 ± 5.7%, 9.7 ± 2.9%, and 8.1 ± 5.5%, respectively. Granulocytes constituted the minimum fraction in most samples (4.8 ± 3.4%).

The CD19^+^ B cells were further classified into four subphenotypes (C-16, C-17, C-33, and C-34). The C-16 cluster was positive for MHC II, CD19, and CD38, while the C-17 cluster had the same expression pattern, except for the coexpression of Siglec-F. In addition, C-33 and C-34 cells also expressed moderate levels of CD11b. Among the CD3^−^ cells, two subphenotypes (C-19 and C-32) were defined as NK cells because of their positive NK1.1 status. The expression of other markers varied between them, as the C-19 cluster was positive for Ki67, CD11c, CD62L, and CD11b, while the C-32 cluster was positive for Siglec-F and CD11b. DCs characterized by CD11c + MHCII+ were identified into three subphenotypes (C-20, C-22, and C-24). C-24 cells expressed high levels of Ki67 and CD103, whereas C-20 and C-22 cells were negative for these markers. For the Ly6G/C^+^ granulocytes, three subphenotypes were defined as eosinophils (C-36 and C-37) and neutrophils (C-38) according to the expression status of Siglec-F. All three clusters were strongly positive for CD11b expression. Ly6C^+^CD11b^+^ monocytes also had four subphenotypes (C-11, C-14, C-15, and C-23). The C-11 and C-23 clusters characteristically expressed CD140a and CD11c, respectively. Clusters C-14 and C-15 had the same expression pattern, including Siglec-F, CD38, and CD31, but with markers expressed at different levels.

### 3.3. In-Depth Analysis of the Dominant Immune Phenotypes

**Figure 2:**
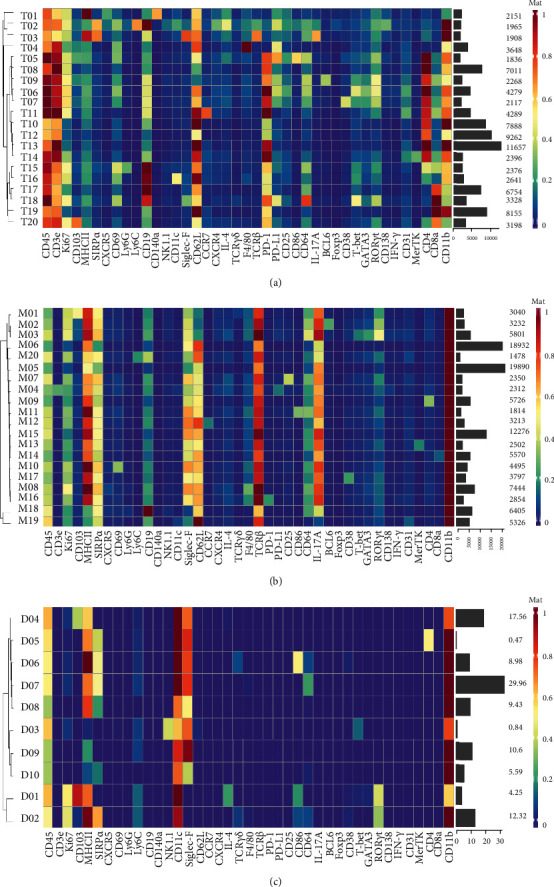
Identification of the dominant immune components in nephrolithiasis models. (a) Heatmap showing normalized expression of the 42-antibody panel markers for the 20 T cell clusters identified with PhenoGraph. (b) Heatmap showing normalized expression of the 42-antibody panel markers for the 20 macrophage clusters identified with PhenoGraph. (c) Heatmap showing normalized expression of the 42-antibody panel markers for the 10 DC clusters identified with PhenoGraph. Clusters are grouped by expression profile with the relative frequencies that are displayed as a bar graph on the right.

As the subsets of T cells, macrophages, and DCs are the dominant immune phenotypes, additional t-SNE and PhenoGraph analyses were performed to exhaustively map these cell phenotypes. These approaches identified the expression profiles of T cells as indicated in the following: 10 CD4^+^ phenotypes, four CD8^+^ phenotypes, one CD4/CD8 double-positive phenotype, and five double-negative phenotype (Figure 2(a) and Supplementary Figure 2A). We observed highly similar phenotypes among CD4^+^ and CD8^+^ subsets, including cells expressing Ki67, Ly6C, Siglec-F, and TCR*β*. The subset of CD4^+^ helper T cells further consisted of clusters such as Treg (T07), Th1 (T06), Th2 (T05), and Th17 (T09). Five populations of CD3+ cells but CD4/CD8 double-negative cells were noted and were predominantly defined by the presence of Siglec-F and absence of TCR*β*. However, some clusters also showed different features, such as the positive expression of PD-1, NK1.1, and TCR*γδ*, indicating exhaustion of T cells (T02), NKT cells (T16), and *γδ*T cells (T04).

The identification of 20 macrophage phenotypes indicated that the macrophages had more subtle differences in marker expression than T cells (Figure 2(b) and Supplementary Figure 2B). The PhenoGraph analysis confirmed a characteristic panel of markers with varied expression levels, including MHC II, SIRP*α*, CD11c, Siglec-F, F4/80, CD64, and CD11b. However, some macrophages may form a spectrum of phenotypically related cell subsets. For instance, M01, M02, and M03 were the only macrophage clusters with high levels of ROR*γ*t expression. Furthermore, M18 and M19 expressed relatively low levels of MHC II and CD11c compared to the other macrophage clusters. For subsets of DCs, a highly similar phenotype of Siglec-F expression was observed in addition to characteristic markers such as MHC II and CD11c (Figure 2(c) and Supplementary Figure 2C).

### 3.4. Correlation Analysis of Intrarenal Resident Immune Components

**Figure 3:**
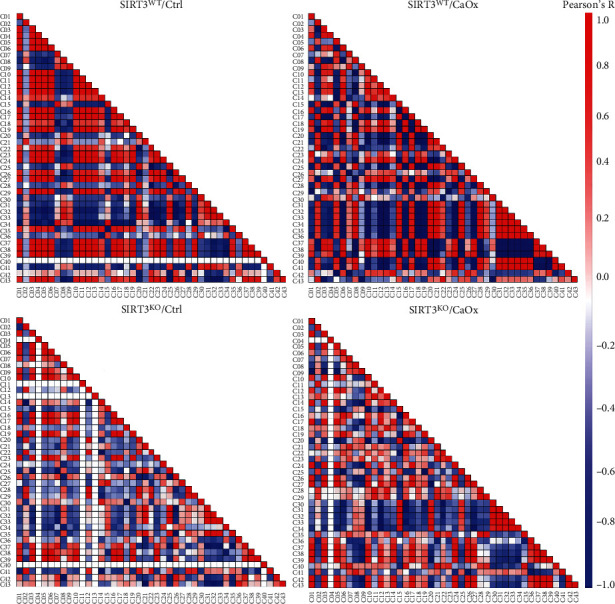
Heatmaps showing Pearson coefficients of correlation for relationships between immune cell phenotypes in four different models with SIRT3 wild-type or knockout and CaOx inducement or not.

To systematically quantify the relationships between resident immune cell populations present in the kidney, we calculated the frequencies for each immune cell phenotype (Figure 3). Multiple robust relationships were first identified and presented in SIRT3 wild-type and CaOx-untreated models. Significant negative correlations were found between certain clusters (C07-09, C15, C20, C30-34, and C41) and the other clusters, indicating that they might be exposed to a similar milieu and tend to follow similar polarization schemes. In addition, the correlations between certain clusters (C02, C05, C17, C22, and C29) and the remaining clusters were reversed under CaOx inducement. However, the same correlation change induced by CaOx was not observed after SIRT3 knockout.

**Figure 4:**
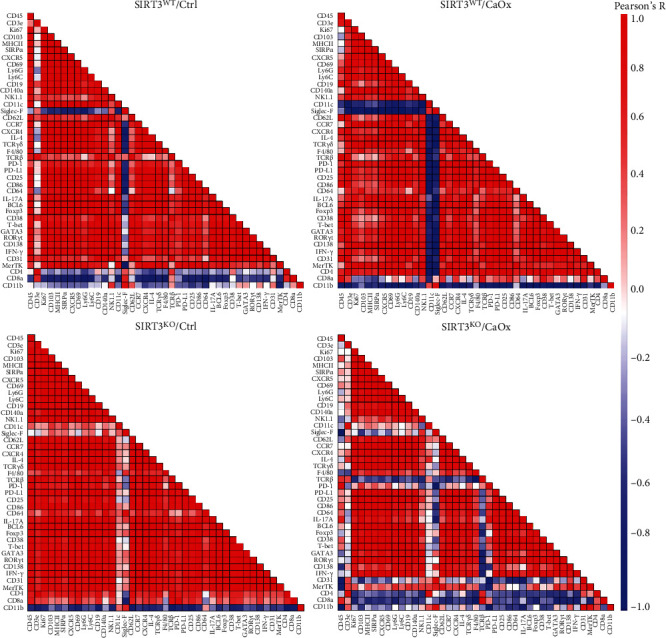
Heatmaps showing Pearson coefficients of correlation for relationships between expressed markers in four different models with SIRT3 wild-type or knockout and CaOx inducement or not.

The influence of SIRT3 knockout and/or CaOx inducement on the frequency correlation of each expressed marker was also analyzed (Figure 4). Except for Siglec-F, CD11b, CD4, and CD8a, the majority of the 42 markers were positively correlated with each other in SIRT3 wild-type and CaOx-untreated renal samples. Under CaOx inducement, the expression of CD4 and CD8a was positively correlated with that of most markers. In contrast, the expression of more markers such as CD31 and TCR*β* showed negative correlations with those of other markers after SIRT3 knockout combined with CaOx inducement.

### 3.5. Comparative Analysis of Immune Landscape between Nephrolithiasis Models

**Figure 5:**
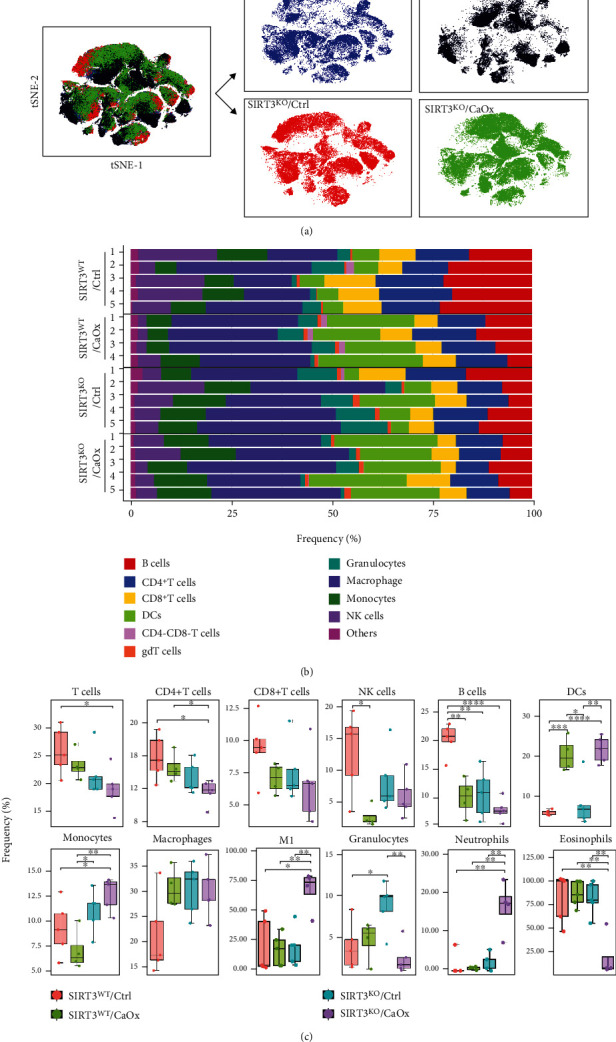
Immune cell population changes after SIRT3 knockout and CaOx inducement. (a) t-SNE maps displaying 9935 cells analyzed with 42-antibody panel and colored by different models. (b) Frequencies of 11 intrarenal immune cell populations for each nephrolithiasis sample. Cell types are indicated by color. (c) Boxplots showing the frequencies of indicated immune cell clusters among four different models with SIRT3 wild-type or knockout and CaOx inducement or not. ^∗^*P* < 0.05, ^∗∗^*P* < 0.01, ^∗∗∗^*P* < 0.001.

First, we applied viSNE to the immune landscape of four different groups, including SIRT3 wild-type or knockout, CaOx inducement, or noninducement (Figure 5(a)). The resulting t-SNE maps showed several differences in densities of particular localized regions, implying altered relative abundances of immune cell types and their subsets. The SIRT3^KO^/CaOx and SIRT3^WT^/CaOx groups exhibited global differences in the intrarenal immune landscape, whereas only small differences were observed between the SIRT3^KO^/CaOx and SIRT3^KO^/Ctrl groups. This indicates that in the CaOx-induced nephrolithiasis model, SIRT3 has a critical role in regulating the immune system, especially in reducing inflammatory injury. When the frequencies of immune lineages were further analyzed for each individual sample (Figure 5(b)), the SIRT3^KO^/Ctrl group displayed the widest variability of immune cell population among samples, followed by the SIRT3^WT^/Ctrl group. However, regardless of SIRT3 knockout, the CaOx-induced nephrolithiasis models had a small heterogeneity of immune cell types within its samples. This indicated that the influence of CaOx on the intrarenal immune components of each individual was consistent.

**Figure 6:**
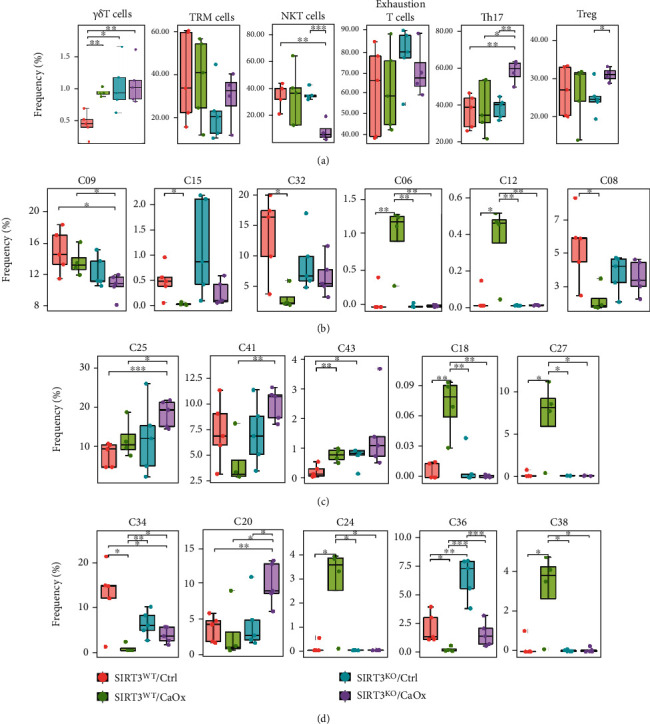
Subphenotypes of (a, b) T cells, (c) macrophages, and (d) other immune cell populations dominantly affected by SIRT3 knockout and CaOx inducement. ^∗^*P* < 0.05, ^∗∗^*P* < 0.01, ^∗∗∗^*P* < 0.001.

As shown in Figure 5(c), among the major immune lineages, the response of CD4^+^ T cells, NK cells, monocytes, M1, neutrophils, and eosinophils to CaOx inducement was regulated by SIRT3 expression. Furthermore, B cells and granulocytes with/without CaOx inducement were also altered by the SIRT3 genetic status. However, the response of B cells, DCs, and granulocytes to CaOx inducement was not influenced by SIRT3 expression, as the degree of expression changes was identical in SIRT3^WT^ and SIRT3^KO^ models. Interestingly, no significant changes in the cell abundance profile of CD8^+^ T cells and macrophages were detected after either CaOx inducement or SIRT3 knockout. As shown in Figure 6(a), a significantly higher abundance of both Th17 and Treg cells and a lower abundance of NKT cells were found in the SIRT3^KO^/CaOx group. No statistically significant difference was found in frequency ratios of Th17 and Treg cells (1.43 ± 0.62 vs. 1.85 ± 0.18 vs. 1.43 ± 0.30 vs. 1.56 ± 0.46) among the four groups. However, upregulated abundance of *γδ*T cells was seen in both SIRT3^KO^/CaOx and SIRT3^WT^/CaOx groups. To further explore which subphenotypes were the dominantly affected, the cell abundances of the clusters identified by our markers were also analyzed (Figures 6(b)–6(d), Supplementary Figure 3). Among the four subtypes of T cells, three clusters (C09, C06, and C12) were found to be significantly lower in abundance in SIRT3^KO^/CaOx relative to the SIRT3^WT^/CaOx group, whereas the remaining one (C08) was more abundant in the SIRT3^KO^/CaOx group (Figure 6(b)). Regarding the many subtypes of macrophages, there were three clusters (C25, C41, and C43) that were found to have significantly elevated abundance in SIRT3^KO^/CaOx relative to the SIRT3^WT^/CaOx group, whereas two clusters (C18 and C27), whose abundance changed in the opposite sense (Figure 6(c)). In addition, the expression difference could also be significantly detected in subphenotypes of immune cells, including B cells (C34), DCs (C20, C24), and granulocytes (C36, C38) (Figure 6(d)).

### 3.6. Expression Patterns of Immune Markers Associated with Nephrolithiasis Risk

**Figure 7:**
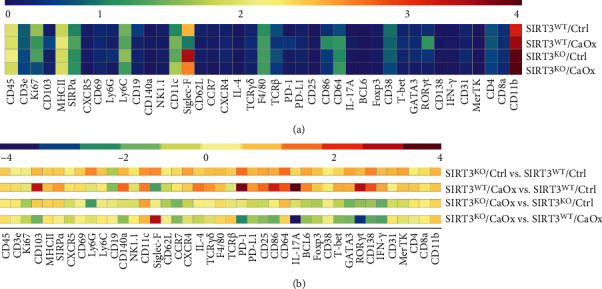
Expression patterns of immune markers associated with nephrolithiasis risk. (a) Heatmap showing the normalized median expression of indicated markers in immune cell populations of four different models with SIRT3 wild-type or knockout and CaOx inducement or not. (b) Heatmaps displaying the relative expression changes of indicated markers in immune cell populations between any two of the four different models.

Using CytoClusterR, the heterogeneity of the 42 detected marker signatures from four different groups, including SIRT3 wild-type or knockout, CaOx inducement, was clearly revealed on heatmaps (Figure 7(a)). Additionally, the normalized mean expression of the indicated markers was compared between the two groups (Figure 7(b)).

The comparison between SIRT3^WT^/Ctrl and SIRT3^KO^/Ctrl models indicated that SIRT3 knockout alone without CaOx inducement had little effect on the expression levels of the markers from immune cells. In contrast, SIRT3 knockout upregulated Siglec-F and downregulated IL-17A, ROR*γ*t, and PD-1 levels in the CaOx inducement groups. Under the two situations with SIRT3 knockout or not, the panel of markers influenced by CaOx inducement varied to a great extent. In the SIRT3^WT^/CaOx group, the expression of PD-1, IL-17A, ROR*γ*t, and CD103 was higher, while expression of CD19, Siglec-F, and CD62L was lower than that in the SIRT3^WT^/Ctrl group (Supplementary Figure 5). However, in the SIRT3^KO^/CaOx group, the expression of most markers such as Ly6G, CCR7, GATA3, and IFN-*γ* was lower than those in the SIRT3^KO^/Ctrl group.

## 4. Discussion

As a cutting-edge, single-cell technology, CyTOF permits high multiparametric measurement of up to 50 metal isotope tags on a single cell simultaneously [13]. This innovation has been applied to understand the heterogeneity and complexity of cellular development [14], differentiation [15], and tumor immunology [16]. Our study is the first to identify distinct immune cell abundance profiles associated with nephrolithiasis through CyTOF-based immunophenotyping. A better understanding of the mechanisms relating the immune system and the underlying renal injury caused by SIRT3 knockout and CaOx inducement could provide novel insights leading to improved prediction and management of nephrolithiasis.

Several recent studies have reported the response of the immune system to CaOx crystals using cell culture and animal models. Dominguez et al. found that monocytes recognize CaOx crystals through a lipopolysaccharide-mediated mechanism, leading to their differentiation into inflammatory M1 macrophages [17]. However, Okada et al. indicated that monocyte-macrophage migration and phagocytosis play roles in the prevention of CaOx crystal formation in mouse kidneys [7]. In addition, two studies demonstrated that both androgen receptor knockout and SIRT3 overexpression could increase renal anti-inflammatory macrophage differentiation and decrease CaOx deposition in mouse models [5, 18]. Intrarenal macrophages participate in both inflammatory and anti-inflammatory processes by phagocytizing antigens in the microenvironment and undergoing phenotypic changes [19]. Overall, these results suggest that the responses of the renal immune system to crystal formation are diverse and dynamic; consequently, the precise function of macrophages in kidney stone formation and elimination could not be determined. Therefore, a better understanding of which macrophages are proinflammatory or anti-inflammatory and the potential interaction mechanism between them will have more practical value.

In-depth immune profiling of this dominant immune phenotype using extensive antibody panels revealed the complexity of macrophage clusters and identified multiple disease-specific subsets. In our study, three of the four macrophage clusters positive for M1 markers CD86 (C27, C29, and C39) were found to be elevated by CaOx inducement, indicating that CaOx promoted M1 macrophage development. Furthermore, the other three clusters (C25, C28, and C41) were elevated by CaOx inducement only in SIRT3 knockout, suggesting that their inflammatory capabilities were suppressed by SIRT3. One cluster (C30) was regarded as a possible anti-inflammatory macrophage, because the abundance was downregulated after CaOx inducement. A genome-wide analysis of the CaOx nephrolithiasis model demonstrated in genetic level that immune reactivity through macrophage migration was involved in both calculi formation and elimination in mouse kidneys [20]. Further *in vivo* and *in vitro* studies have shown that M1 and anti-inflammatory M2 macrophages have opposing roles in nephrolithiasis [6]. In summary, these suggest the potential of immune-based therapies for urinary calculi.

Our study indicated that MHC II, SIRP*α*, CD11c, Siglec-F, F4/80, CD64, and CD11b were the characteristic panels of markers for interstitial macrophages. Other studies have also reported changes in the expression level and potential biological capabilities of these immune molecules. During CaOx crystal formation, MHCII was immunohistochemically upregulated around crystal formation sites along with an increase in interstitial macrophages. Furthermore, the association analysis of the related gene expression by RT-PCR indicated a high association of CCL2, CD44, CSF-1, SPP-1, fibronectin 1, and TGF-*β*1 with the amount of both renal crystals and F4/80, a mouse macrophage marker [7]. Interestingly, the absolute number of renal interstitial macrophages did not increase constantly but fluctuated with the glyoxylate-induced crystal deposition process.

In the innate immune response, helper T cells play a prominent role by recognizing self-antigens, regulating cytokine production, and inducing humoral immunity [21]. As the two main subsets of helper T cells, Th1 and Th2 cells are regarded as inflammatory and anti-inflammatory helper T cells, respectively. Hsi et al. reported that Th1 cells drove the proinflammatory and Th2 anti-inflammatory responses in atherosclerosis, which have similar calcification lesions on vascular endothelial cells as urolithiasis [22]. In this study, we discovered that cluster T06, belonging to Th1, was more abundant in the CaOx inducement model than in the control model, although the difference was not statistically significant. ROR*γ*t^+^IL-17^+^ Th17 and FOXP3^+^CD25^+^ Treg cells are critical subsets of CD4^+^ T cells that are essential in immune homeostasis. The transcription factor FOXP3 is essential for Treg cell development and function (inhibition and suppression, self-tolerance), whereas transcription factor ROR*γ*t is also essential for Th17 cells (induction and propagation, tissue inflammation) [23]. The frequency intervention of Th17/Treg cells may provide new insights into the therapeutic targets of nephrolithiasis. In this study, we demonstrated that cluster T09 belonged to Th17, while cluster T07 belonged to Treg, and the ratio of T09/T07 was significantly increased after CaOx inducement.

In normal mouse kidneys, CD4/CD8 double-negative T cells were shown to comprise a higher percentage of the T cell population than those in other organs such as the liver and lungs [24]. Furthermore, CD4/CD8 double-negative T cells were discovered to have a pathogenic role in early renal injury after ischemia-reperfusion injury (IRI) [25]. *γδ*T cells are a minor subset of T cells and are often thought to form a bridge between innate and adaptive immunity. Savransky et al. suggested that deficiency of *γδ*T cells protected the kidney from IRI to a similar extent as deficiency of *αβ*T cells [26]. In our study, the intrarenal *γδ*T cell population was found to be increased in CaOx-induced nephrolithiasis compared to controls. Targeting this subphenotype with additional markers will be of interest in future research. NKT cells belong to a unique lymphocyte population expressing both NK receptors and TCRs and exert regulatory functions by secreting cytokines such as IL-4, IL-10, IFN-*γ*, and NK1.1 [25]. However, NKT cells play conflicting roles in the process of renal injury. Li et al. showed that NKT cells contribute to the induction of early renal injury by mediating neutrophil IFN-*γ* production [27]. Another report by Yang et al. indicated that NKT cells, especially type II NKT cells, attenuated the severity of renal injury [28].

In this study, we discovered that the expression of Siglec-F and CD11b was negatively correlated with all the other markers in intrarenal resident immune cell populations. Siglec-F is conveniently used as a cell-specific marker of eosinophils. A recent study by Tateyama et al. showed that the expression of Siglec-F in bone marrow-derived macrophages could be stimulated by granulocyte-macrophage colony-stimulating factor (GM-CSF) first and then downregulated upon prolonged GM-CSF stimulation. Furthermore, Siglec-F positively regulated the STAT6 signaling pathway as well as the expression of arginase-1 in IL-4-stimulated macrophages. These results suggested that Siglec-F was induced by GM-CSF and fine-tuned macrophage responses [29]. Alexander et al. suggested that knockout of CD11b on mononuclear cells could recruit more M1 macrophages and CD4^+^ T cells in glomerulonephritis, indicating that CD11b is instrumental in generating an anti-inflammatory response in the inflamed kidney [30]. Kitagawa et al. suggested that urinary CD11b might be a useful biomarker to estimate histopathological activity, particularly glomerular leukocyte accumulation, in lupus nephritis [31]. In addition, while renal tissue is not amenable to regular sampling, the peripheral immune system is easily accessible and well-suited for routine measurement. Therefore, the future of identifying high-risk populations with nephrolithiasis and predicting the recurrence possibility seems to rely on a panel of multiple urinary or peripheral immune markers.

The compelling evidence from our study and others demonstrates the role of the immune system in mediating renal CaOx calculi formation and pathogenesis. Thus, modulating the immune response, such as promoting M2 over M1 macrophages and inhibiting inflammation, might provide a means to prevent CaOx nucleation and renal injury. Investigating the relationship between the immune system and calculi disease may lead to a new era of nephrolithiasis [32]. Future studies on calculi formation and immune biology will identify immunotherapeutic targets for the treatment and prevention of nephrolithiasis [33].

With five individual samples (four in the SIRT3^WT^/CaOx group) in each mouse model, our study was able to identify a number of immune cell subsets demonstrating altered abundance in CaOx inducement models relative to controls and in SIRT3 knockout models compared to wild-type models. However, the relatively small sample size could not eliminate the average variations that could hide high interindividual variations. Furthermore, the mechanism by which these altered immune cell types and their subsets are involved in the pathogenesis of SIRT3 knockout and CaOx-induced renal injury remains unclear. Lastly, further investigations are warranted to include a wider range of cell type-specific markers and more clinical samples to verify the novel biomarkers and immunotherapeutic targets for nephrolithiasis.

## 5. Conclusions

In summary, our research is the first to present a CyTOF-based atlas of the immune landscape in nephrolithiasis models to better understand how the immune system contributes to, and is affected by, the underlying renal injury caused by SIRT3 knockout and CaOx inducement. The data indicated that SIRT3 plays a critical role in regulating the immune system, especially in reducing inflammatory injury, in the CaOx-induced nephrolithiasis model. The characteristic panel of changed immune clusters and markers will provide novel insights leading to improved prediction and management of nephrolithiasis.

## Data Availability

The authors can make data available on request through contacting the corresponding authors.

## Supplementary Materials

Supplementary 1 Supplementary Figure 1: (A) Experimental approach used in this study. (B) Von Kossa staining and immunohistochemical staining to confirm the efficiency of SIRT3 knockout and CaOx inducement. (C) Cell numbers and vitalities of single-cell suspension detected using a CyTOF system. (D) Markers used to characterize the immune cell phenotypes.

Supplementary 2 Supplementary Figure 2: (A) t-SNE map displaying 2096 cells from T cell clusters identified with PhenoGraph colored by cluster. (B) t-SNE map displaying 2792 cells from macrophage clusters identified with PhenoGraph colored by cluster. (C) t-SNE map displaying 1361 cells from DC clusters identified with PhenoGraph colored by cluster.

Supplementary 3 Supplementary Figure 3: complete presentation of immune cell subphenotype changes after SIRT3 knockout and CaOx inducement in all the intrarenal immune cells.

Supplementary 4 Supplementary Figure 4: complete presentation of immune cell subphenotype changes after SIRT3 knockout and CaOx inducement in (A) T cells, (B) macrophages, and (C) DCs.

Supplementary 5 Supplementary Figure 5: t-SNE maps displaying the most significant differentially expressed markers among four different models with SIRT3 wild-type or knockout and CaOx inducement or not.
