# Supplementary material for: An Immune Atlas of Nephrolithiasis: Single-Cell Mass Cytometry on SIRT3 Knockout and Calcium Oxalate-Induced Renal Injury
Source: J Immunol Res. 2021 Nov 20;2021:1260140. doi: 10.1155/2021/1260140 (PMC8627562; doi:10.1155/2021/1260140)
Supplement: Supplementary 1 — Supplementary Figure 1: (A) Experimental approach used in this study. (B) Von Kossa staining and immunohistochemical staining to confirm the efficiency of SIRT3 knockout and CaOx inducement. (C) Cell numbers and vitalities of single-cell suspension detected using a CyTOF system. (D) Markers used to characterize the immune cell phenotypes. [file 1260140.f1.pdf]

A

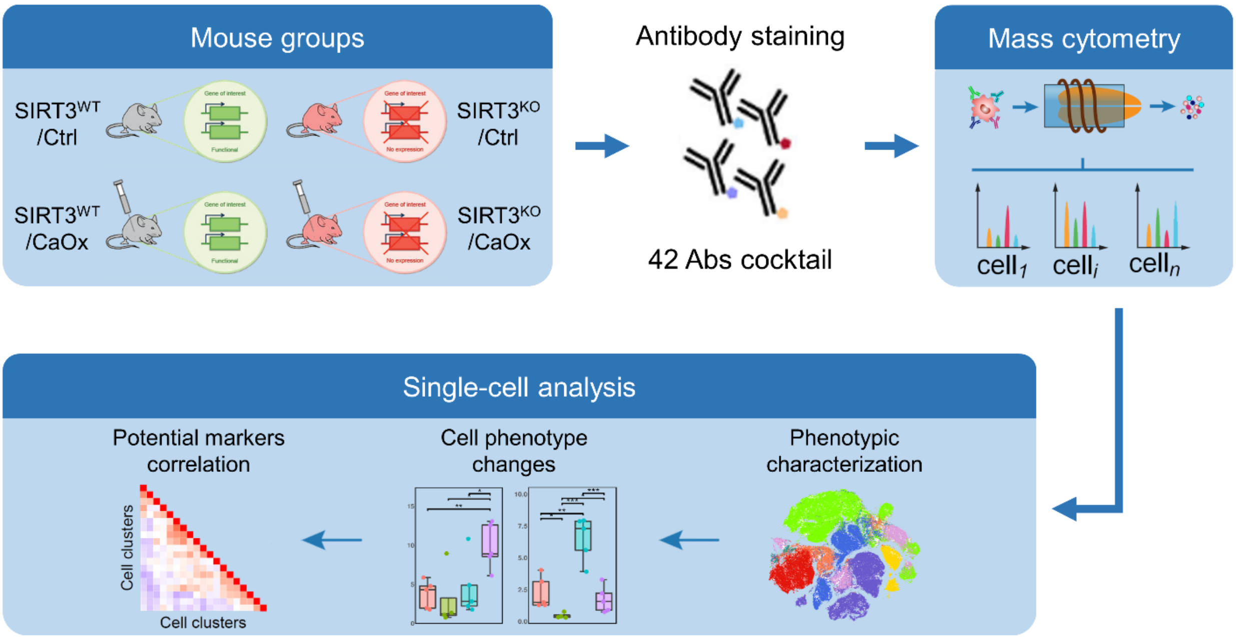

B

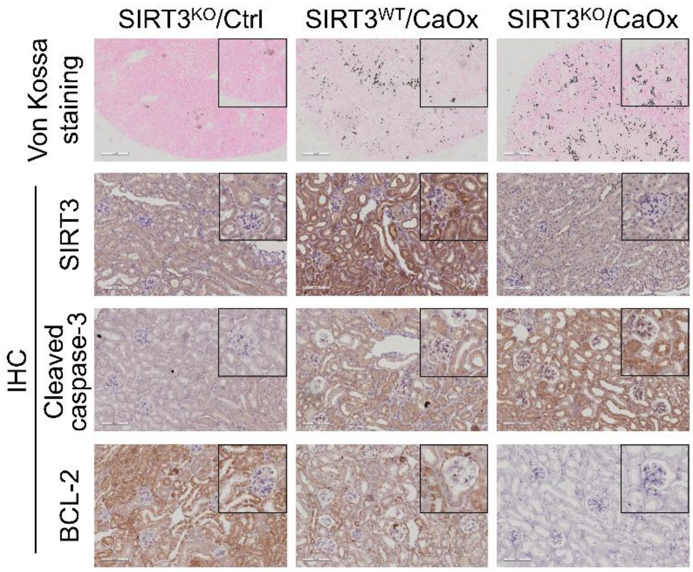

C

Group	Cell number (×10^6)	Vitality (%)
SIRT3^{WT}/Ctrl	1.53	62.7
	1.62	42.0
	2.14	43.2
	3.22	32.7
	1.97	39.4
SIRT3^{WT}/CaOx	1.84	42.4
	1.47	39.9
	2.18	40.0
	2.65	41.8
SIRT3^{KO}/Ctrl	1.30	49.8
	2.84	28.4
	3.57	26.4
	2.09	31.9
	1.62	42.4
SIRT3^{KO}/CaOx	5.31	36.8
	2.21	46.8
	2.01	37.4
	3.75	43.7
	3.03	49.8

D

42 Abs cocktail					
CD45	CD69	CD11c	F4/80	IL-17A	CD138
CD3e	Ter119	Siglec-F	TCRβ	BCL6	IFN-γ
Ki67	Ly6G	CD62L	PD-1	Foxp3	CD31
CD103	Ly6C	CCR7	PD-L1	CD38	MerTK
MHCII	CD19	CXCR4	CD25	T-bet	CD4
SIRPα	CD140a	IL-4	CD86	GATA3	CD8a
CXCR5	NK1.1	TCRγδ	CD64	RORγt	CD11b
