# Supplementary figures and images for: An Immune Atlas of Nephrolithiasis: Single-Cell Mass Cytometry on SIRT3 Knockout and Calcium Oxalate-Induced Renal Injury
Source: J Immunol Res. 2021 Nov 20;2021:1260140. doi: 10.1155/2021/1260140 (PMC8627562; doi:10.1155/2021/1260140)

A

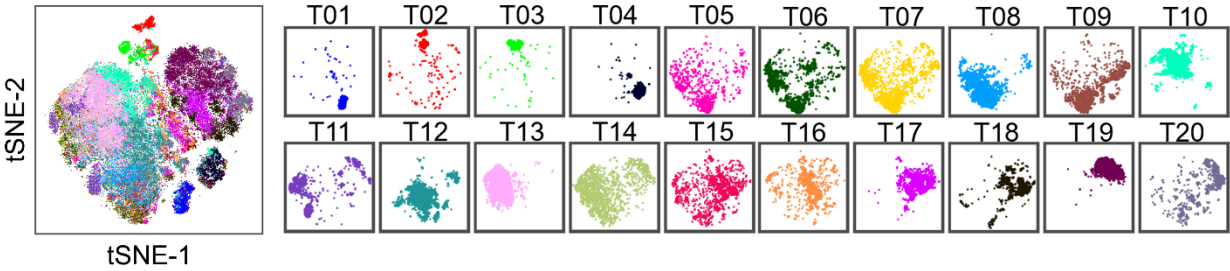

B

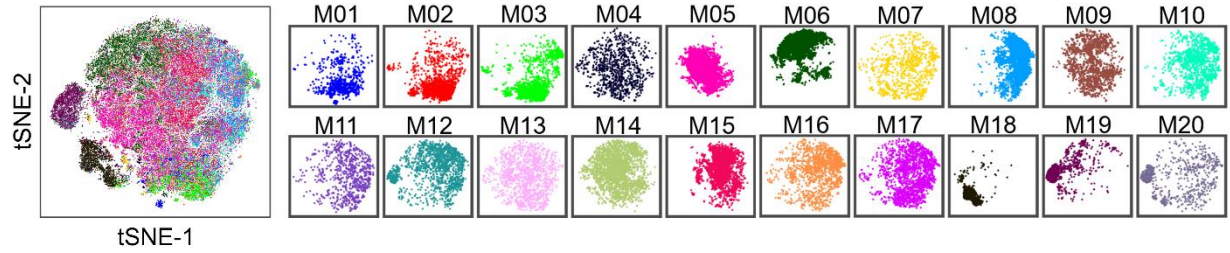

C

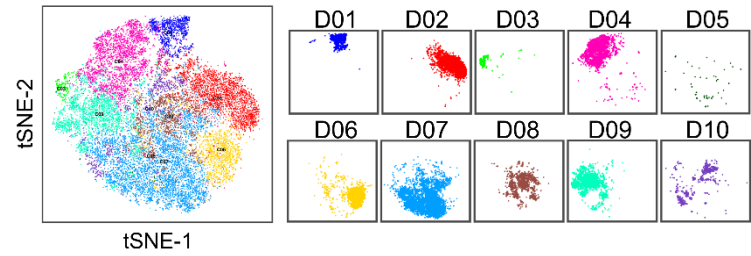

Supplement: Supplementary 2 — Supplementary Figure 2: (A) t-SNE map displaying 2096 cells from T cell clusters identified with PhenoGraph colored by cluster. (B) t-SNE map displaying 2792 cells from macrophage clusters identified with PhenoGraph colored by cluster. (C) t-SNE map displaying 1361 cells from DC clusters identified with PhenoGraph colored by cluster. [file 1260140.f2.pdf]

Frequency (%)

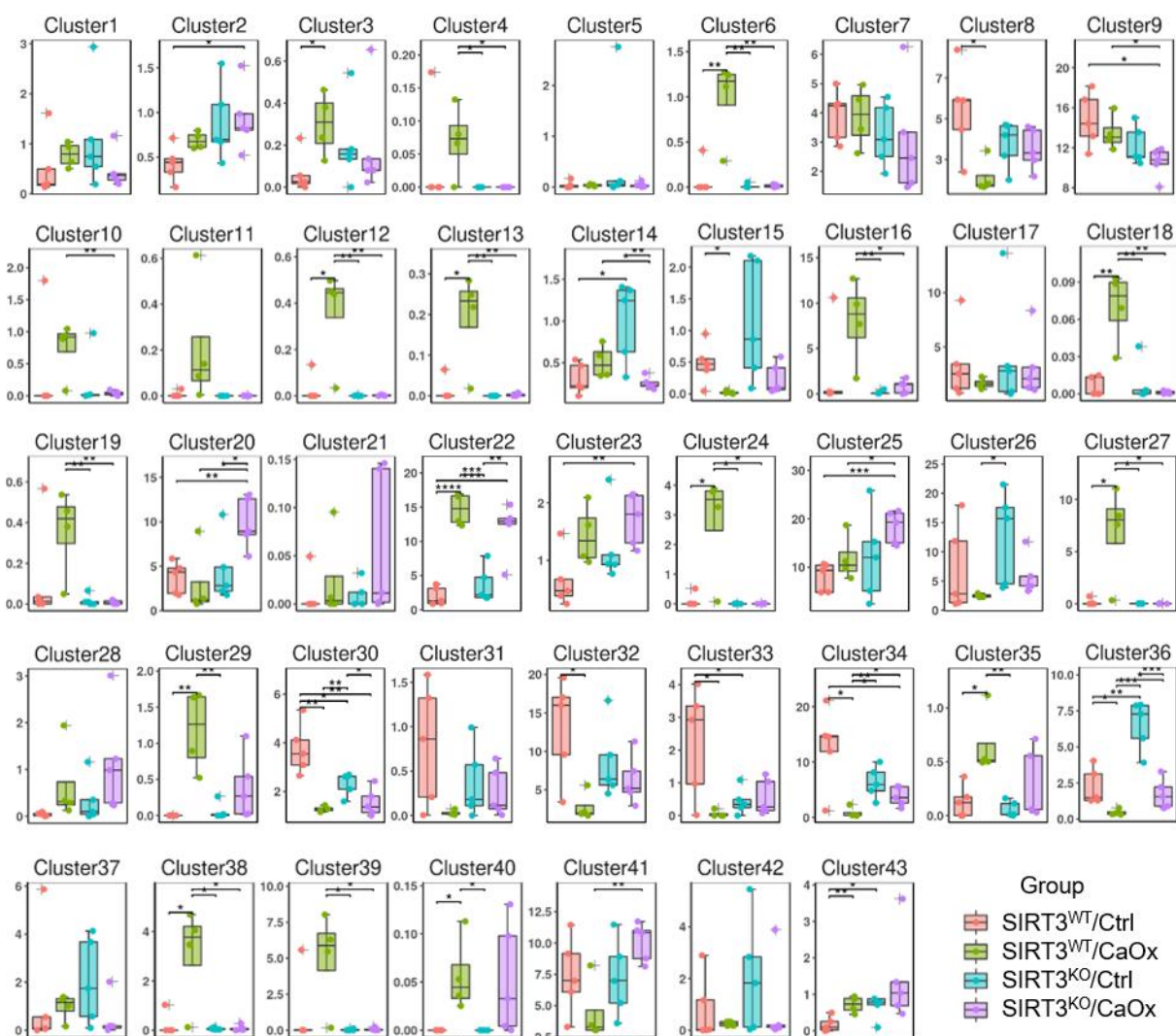

Supplement: Supplementary 3 — Supplementary Figure 3: complete presentation of immune cell subphenotype changes after SIRT3 knockout and CaOx inducement in all the intrarenal immune cells. [file 1260140.f3.pdf]

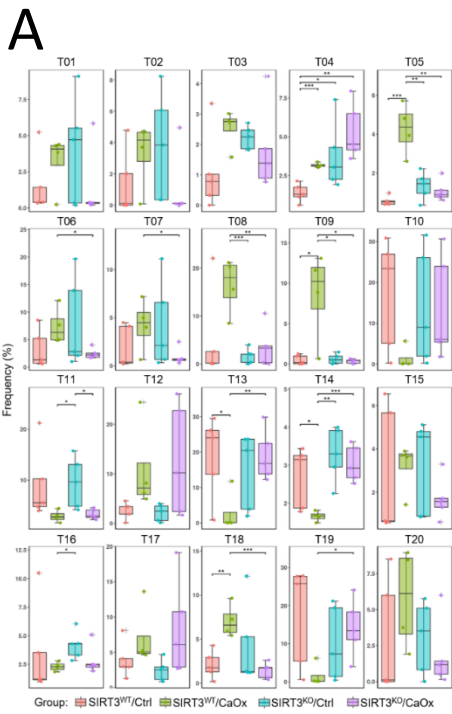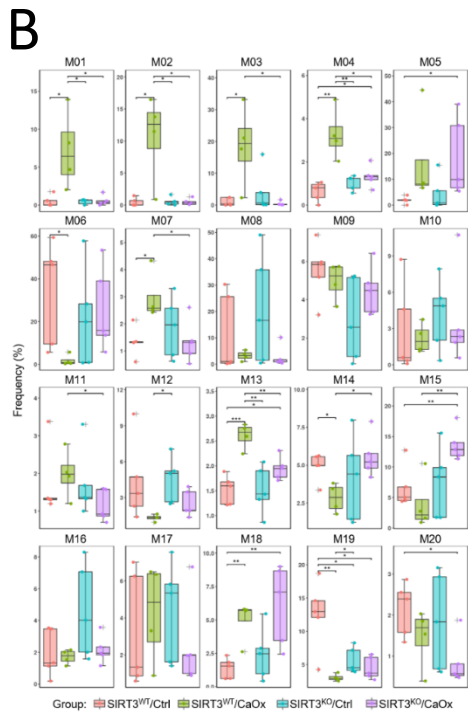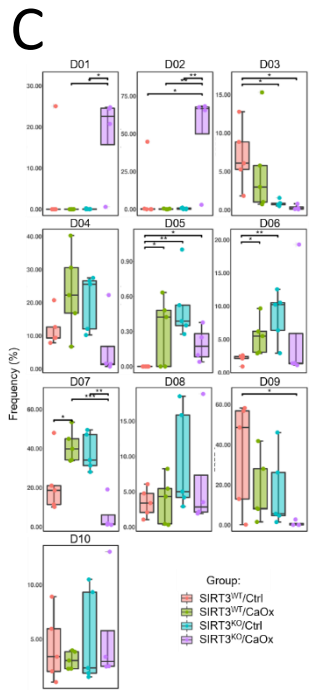

Supplement: Supplementary 4 — Supplementary Figure 4: complete presentation of immune cell subphenotype changes after SIRT3 knockout and CaOx inducement in (A) T cells, (B) macrophages, and (C) DCs. [file 1260140.f4.pdf]

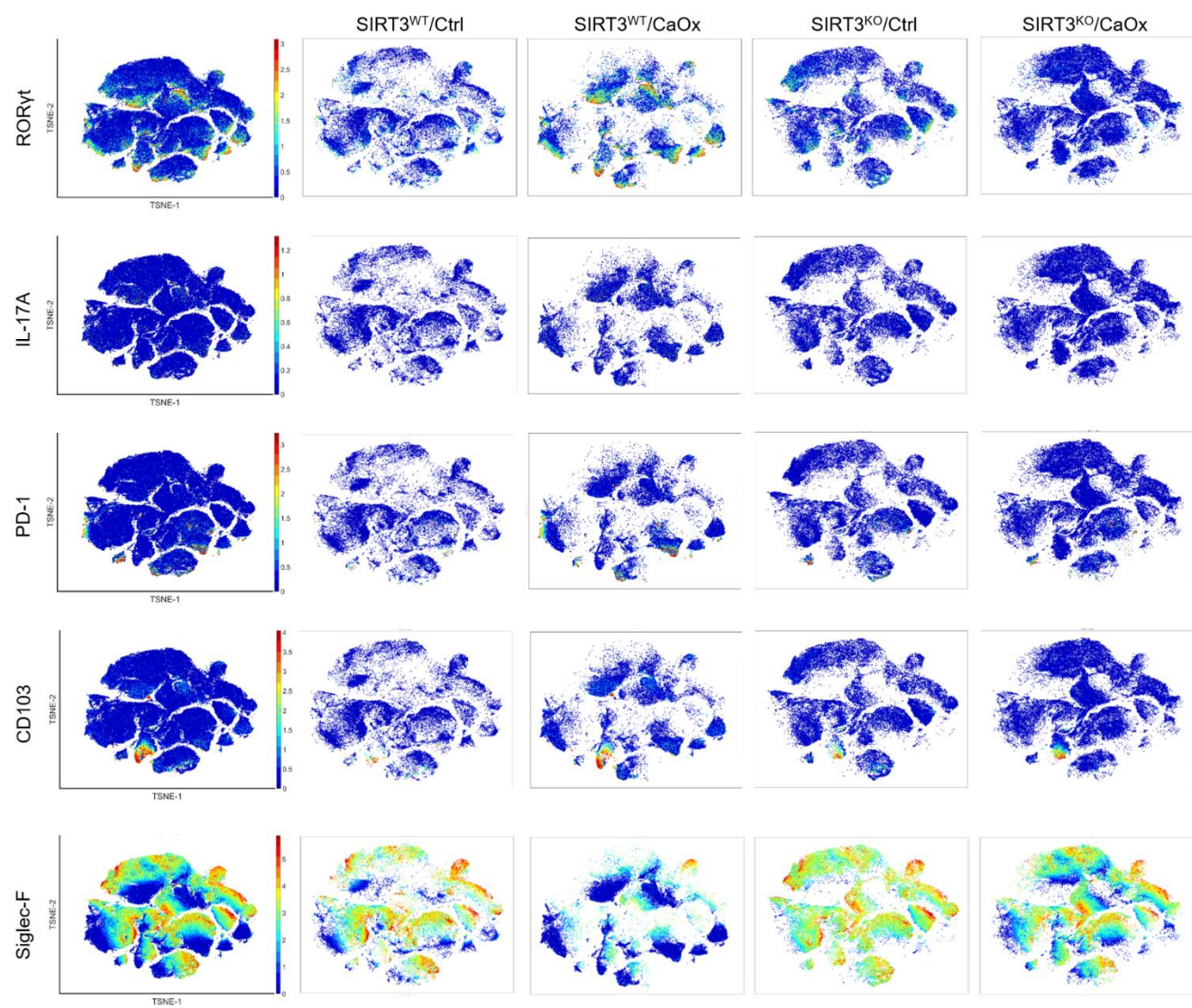

Supplement: Supplementary 5 — Supplementary Figure 5: t-SNE maps displaying the most significant differentially expressed markers among four different models with SIRT3 wild-type or knockout and CaOx inducement or not. [file 1260140.f5.pdf]
